# TNF-α - mediated peripheral and central inflammation are associated with increased incidence of PND in acute postoperative pain

**DOI:** 10.1186/s12871-021-01302-z

**Published:** 2021-03-17

**Authors:** Yu-fan Zhao, Hui-wen Yang, Ting-shun Yang, Wenxiu Xie, Zhong-hua Hu

**Affiliations:** 1 Department of Anaesthesiology, The Third Xiangya Hospital, Central South University, 138 Tongzipo Road, Changsha, Hunan 410013 People’s Republic of China; 2 Department of Anaesthesiology, Hunan Cancer Hospital, Central South University, Changsha, 410013 Hunan People’s Republic of China

**Keywords:** Perioperative neurocognitive disorders (PND), Acute postoperative pain, TNF-α, Microglia, C-Fos

## Abstract

**Background:**

Acute postoperative pain plays an important role in the perioperative neurocognitive disorders (PND). The pathogenesis of PND is still unknown, but it is generally believed that peripheral and central nervous system inflammation play an important role, and acute postoperative pain is also thought to aggravate postoperative inflammatory response. The aim of the present study is to explore the effect of acute postoperative pain on peripheral and central nervous system inflammation and related cognitive impairment behaviour in elderly rats after surgery.

**Methods:**

Rats were assigned into four groups: control, surgery for internal fixation for tibial fracture, surgery with analgesia using intraperitoneal morphine, and morphine without surgery. Pain was assessed by the Subjective Pain Scale. The spatial memory of rats was assessed by the Morris water maze (delayed matching task) from the second day to the seventh day after surgery (POD2-POD7). In part of the rats, the pro-inflammatory cytokines TNF-α in plasma, the medial prefrontal cortex (mPFC), and the hippocampus were determined by ELISA on the POD2. The activation of microglia and the expression of c-Fos in the hippocampal CA1 regions and mPFC were detected by the immunohistochemical method on the POD2.

**Results:**

Acute postoperative pain and spatial memory impairment occurred after operation, and postoperative analgesia could significantly improve the both parameters. Additionally, on the POD2, the levels of TNF-α in plasma, hippocampus and mPFC were significantly increased, while the activation of microglia cells and the expression c-Fos in the hippocampal CA1 regions and mPFC were significantly increased. And postoperative analgesia with morphine significantly inhibited the above reactions.

**Conclusion:**

Our data suggest that acute postoperative pain increases the incidence of perioperative neurocognitive disorders. Peripheral and central nervous system inflammation may be involved in this cognitive impairment. And reducing the intensity of acute postoperative pain may be one of the main preventive strategies for PND.

## Background

Perioperative Neurocognitive Disorders (PND) is a standardized name for anaesthesia- and operation-related cognitive disorders given in 2018 by a multidisciplinary expert group to replace the original term Postoperative Cognitive Dysfunction (POCD) [1]. PND is a common complication in the perioperative period of elderly patients, with an incidence of about 11.7–60%, mainly reflected by changes in personality, social ability, and cognitive ability, specifically manifested as slow reaction, inattention, impaired memory and learning, and difficulty in initiating and executing commands [2]. The generally recognised risk factors for PND include advanced age, low education level, pre-existing cognitive impairment, type of surgery (cardiac surgery, orthopaedic surgery, vascular surgery), postoperative respiratory complications, postoperative infection, and uncontrolled postoperative pain [3]. Among them, the influence of acute postoperative pain on PND has attracted increasing attention. Studies have shown that adequate postoperative analgesia can reduce the incidence of PND after femoral fracture surgery in elderly patients [4]. Preclinical and human experiments indicate that peripheral and central nervous system (CNS) inflammation play an important part in the progression of PND [5–7]. However, the pathogenesis of PND has not been fully elucidated.

Postoperative pain is an unpleasant sensation and emotional experience caused by tissue damage during the operation [8]. It can be divided into acute postoperative pain and chronic postoperative pain according to its duration. In the 11th revision of the International Classification of Diseases (ICD) published by the World Health Organization in 2018, acute postoperative pain was defined as immediate acute pain after surgery, lasting less than 1 month. Severe acute postoperative pain is associated with multiple complications, possibly including impaired cognitive function [7]. Moreover, some studies have shown that acute postoperative pain can aggravate the postoperative peripheral system inflammation [4, 9]. However, whether acute postoperative pain exacerbates CNS inflammation and related cognitive impairment behaviour remains unclear.

CNS inflammation mainly involves the activation of microglia cells and the release of pro-inflammatory cytokines such as TNF- α. Microglia are a specialised macrophage population in the CNS, which can be activated by insult factors and are the first cells to induce the neuroinflammatory response [10]. Studies have shown that the activation of microglia cells and the release of inflammatory cytokines in brain regions related to cognitive function are associated with the occurrence of PND [10]. c-fos is a member of the immediate early gene family, and the c-fos protein (c-Fos) is expressed rapidly after neurons are stimulated [11]. Studies have shown that noxious stimuli can induce the upregulation of c-Fos in the central nervous system, including dorsal root ganglion, spinal dorsal horn and related brain regions [11–13]. Therefore, the expression of c-Fos can be used as a marker of response to noxious stimuli in the corresponding brain regions.

The hippocampus and the medial prefrontal cortex (mPFC) are closely related to cognitive function, some studies have shown that pain stimulation can also activate these brain regions [14, 15]. The overlap of related brain areas may provide the anatomical basis for pain exacerbating cognitive impairment. Therefore, in the present study, we verified the role of acute postoperative pain in PND by constructing a tibial fracture model of elderly rats. Then we detected the expressions of TNF-α in plasma, mPFC, and the hippocampus by ELISA. The expression of ionised calcium binding adaptor molecule 1 (Iba1, microglial marker) and c-Fos in the hippocampal CA1 regions and mPFC were assessed by the immunohistochemical. To further explore the effect of acute postoperative pain on peripheral and central nervous system inflammation and related cognitive impairment behaviour in elderly rats after surgery.

## Materials and methods

### Animals and groups

Aged (21–22 months) male Sprague-Dawley rats were obtained from Shanghai Sipur-Bikai Experimental Animal Co. Ltd. The rats were raised under controlled conditions (22–25 °C, 12-h alternate circadian rhythm, food, and water were available ad libitum). All animal studies were approved by the Experimental Animal Care and Use Committee of Central South University (2020sydw0630) and performed to comply strictly with the guidelines accepted by the International Association for the Study of Pain. The rats were randomly divided into four groups (*n* = 18 rats/ group): (C) anaesthesia without surgery (control group); (S) anaesthesia with surgery (surgery group); (S + M) anaesthesia with surgery plus intraperitoneal injection of morphine (surgery+ morphine group); (M) anaesthesia without surgery plus intraperitoneal injection of morphine (morphine group). Immediately after operation to 6 days after operation, morphine was injected intraperitoneally in group M and group S + M for 3 mg/kg, while C group and S group were given intraperitoneal injections of the same amount of saline, with a frequency of about 4 times per day every 6 h. Testers conducting the subsequent experiments were, where possible, blind to group assignments.

### Internal fixation of tibial fracture

Internal fixation of tibial fractures was performed as previously described [16]. Animals received 92% oxygen and 8% sevoflurane anaesthesia induction. Anaesthesia was maintained with 50% oxygen and 3% sevoflurane. During anesthesia, we continuously monitored the heart rate, breath rate and arterial oxygen saturation level non-invasively using small animal pulse oximeter (STARR, USA).

Under aseptic conditions, the tibia shaft was exposed through a 1 cm incision in the skin of the middle and upper left tibia. A 8 mm incision was performed on the tibial plateau, followed by the insertion of a 7.0 mm pin into the intramedullary canal. Then the periosteum was stripped, and osteotomy was created at the junction of the middle and distal thirds of the tibia, under direct vision using scissors. After surgery, skin incisions were sutured and chlortetracycline ointment was applied to the wound to prevent infection. Intraperitoneal injection of normal saline or morphine was given berore awakening.

### Subjective pain scales

**Fig. 1:**
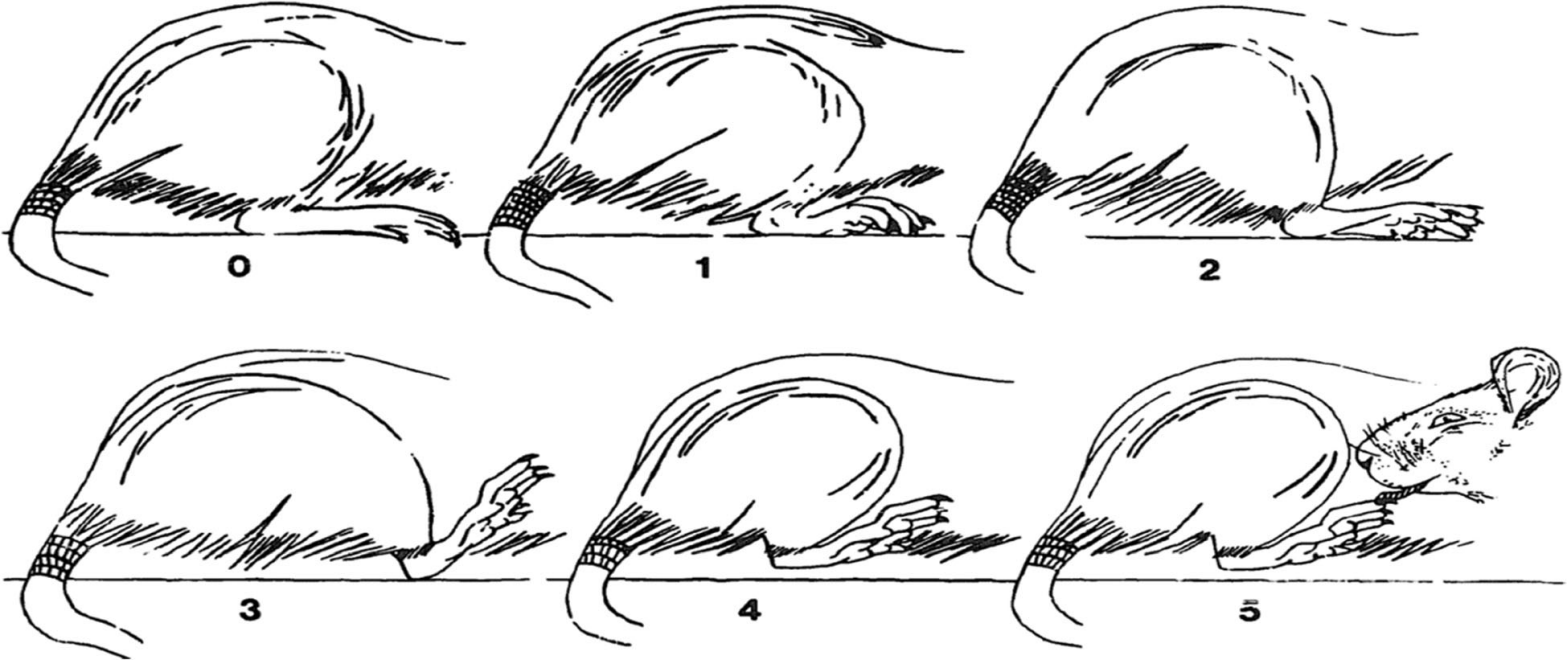
Subjective Pain Scale. The drawing is taken from Attal’s study. The method allows observation without intervention from the experimenter. Six different positions are rated from 0 to 6 in order to quantify the nociceptive reaction [17].

Referring to the methods of Attal et al. [17], the Subjective Pain Scale was adopted to evaluate the pain degree of rats after surgery. On the day of surgery, pain tests were performed at 2 h and 6 h postoperatively. From POD1-POD7, pain tests were performed once a day, at the same time in the morning and 2 h after intraperitoneal injection. The test animals were placed in a plexiglass cage with an 8 mm × 8 mm stainless steel mesh at the bottom. Before the test, the test animals were placed in an adaptive environment for more than 30 min. One animal at a time was used and observed for 300 s. Pain was scored according to the following scale (Fig. 1): 0 = the operated paw is pressed normally on the floor; 1 = the paw rests lightly on the floor and the toes are in a ventroflexed position; 2 = only the internal edge of the paw is pressed on the floor; 3 = only the heel is pressed on the floor and the hind paw is in an inverted position; 4 = the whole paw is elevated; 5 = the animal licks the operated paw. Then the pain score was calculated using the following formula: (t1+ 2 t2+ 3 t3 + 4 t4 + 5 t5)/300 s, where t1, t2, t3, t4 and t5 are the durations spent in categories 1, 2, 3, 4 or 5, respectively.

### Morris water maze (delayed matching-to-place)

The task was conducted in a water maze (Morris, 1984) – a circular pool, 160 cm in diameter and 30 cm height – located in a well-lit room with numerous visual cues. The pool was filled with water at 23 ± 1 °C, made opaque by the black ink. The escape platform was 10 cm in diameter with its top surface 1 cm below the water level so as to be hidden from view at the water surface [18]. The reference memory task is a classic test of spatial memory in Morris water maze. For each trial a rat was gently placed in the pool at one of four locations. Rats were allowed 90 s to locate a 10 cm diameter black platform submerged 1 cm below the surface of the water using visual cues in the room and around the edges of the pool. Each rat was randomly assigned one of four different platform positions, which was kept constant for each individual rat during for the entire test [19]. The delayed matching-to-place (DMP) task differs crucially from the traditional reference memory task in that the platform location varies from day to day [20]. DMP is more difficult because the rats learn to swim to different places every day, which requires flexible use of their “knowledge” of the environment. The rats were trained on the DMP task with four trials per day over a total of 5 days before the surgery. During training, the intertrial intervals (ITI) of trials 1, 2, 3 and 4 were all 15 s. The formal test was carried out for 6 days from the second day after the operation (POD2). In the first 3 days (POD2–4), the ITI between trial 1 and trial 2 was 20 min, while the ITI between trial 1 and trial 2 in the last 3 days (POD5–7) was 2 h. The ITI between trial 2 and trial3 and between trial3 and trial 4 were maintained at a constant 15 s. Starting positions (N, W, S or E) and platform positions (8 different locations; evenly distributed in the pool) were counterbalanced such that they were equally distributed across groups, ITIs and days. In each experiment, a rat was gently placed in one of four positions in a pool, fixed each day. Rats were allowed 60 s to locate the platform using visual cues in the room and around the edges of the pool. After the rats climbed onto the platform, they rested for 15 s for the next experiment. If the rats did not find the platform within 60s, the rats were led to the platform for 15 s and then the next experiment was conducted. Trials were videotaped and swim path was tracked using Ethovision Video-Tracking Software (v3.0 Noldus Information Technology). For each test, the difference of the latency to reach the platform (T1-T2) and path length (S1-S2) between trial 1 and trial 2 and swimming speed were calculated. Larger values of “T1-T2” and “S1-S2” represent better learning and memory.

### Enzyme-linked immunosorbent assay (ELISA)

Two days after the surgery, the rats were decapitated under anaesthesia (pentobarbital sodium, 50 mg/kg, intraperitoneally). The hippocampus, medial prefrontal cortex (mPFC) and blood samples were rapidly harvested. The hippocampal and mPFC samples were homogenized in an ice-soluble buffer containing protease inhibitor and centrifuged at 14000 g at 4 °C for 15 min. The supernatant was stored at − 80 °C for further analysis. The plasma was collected after centrifugation at 2500 g for 10 min and stored at − 80 °C until analysis. The levels of tumour necrosis factor-α (TNF-α) were analysed by the enzyme-linked immunosorbent assay kits (ELISA, Huamei, Wuhan, China), detection limits: 6.25 pg /mL-400 pg/mL, sensitivity: 1.56 pg /mL.

### Immunohistochemistry

Referring to the methods of Wadhwa et al. [21]. The rats were deeply anaesthetised with pentobarbital sodium (50 mg/kg, intraperitoneally), then transcardially perfused with 250-mL ice-cold 0.1 M PBS followed by 4% paraformaldehyde in 0.1 M phosphate buffered saline. The brain tissues were collected and postfixed in 4% paraformaldehyde/phosphate buffered saline overnight and cryoprotected with 30% sucrose/PBS for 48 h at 4 °C. Coronal 20-mm-thick sections were cut on a cryostat (Leica, Wiesbaden, Germany) serially, and the sections of the hippocampus and mPFC were collected from each brain. The sections were placed in 3% hydrogen peroxide for 10 min to eliminate peroxidase in the tissues. After rinsing the sections 3 times with PBS for 10 min, the sections were incubated for 24 h with rabbit anti-Iba1 (1:1000, WAKO, Japan) and rabbit anti-c-Fos (1:2000, Abcam, USA). Then after washing the sections 3 times in PBS, the sections were treated with anti-rabbit secondary antibodies (1:200, Vectastain, USA) for 2 h at room temperature. They were then washed again 3 times in PBS and treated with DAB reagent (Zhongshan Jinqiao, China). Finally, the sections were then affixed to the slides, sealed and dried. Sections were observed under a bright field microscope with × 10, × 20 and × 40 objective lenses, and the images were taken by a photomicrograph system (Nikon, Japan). The morphology of microglial cells (Iba-1) was observed under microscope. The immunoreactivity of proteins (Iba-1, c-FOS) were measured by Image J software.

### Statistical analyses

All data were analysed by SPSS 18.0 and represented as mean ± standard deviation (SD). Differences between the study groups were compared with the single-factor analysis of variance (ANOVA). If the variance was homogeneous, the Bonferroni method was used for multiple comparisons after the event (pairwise comparison of each group), and if the variance was uneven, the Tamhane’s T2 method was used. Statistical figures were drawn using GraphPad prism 7. Statistical significance was indicated by *P* < 0.05.

## Results

### Acute postoperative pain induced by internal fixation of tibial fracture

**Fig. 2:**
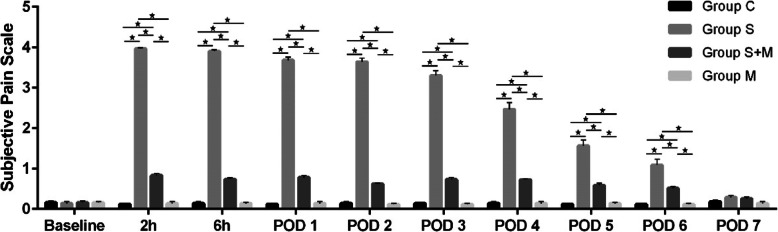
Changes of Subjective Pain Scale scores before and after internal fixation of tibial fracture. From 2 h after surgery, the pain score in group S was significantly higher than that in group C. The overall pain score in group S showed a downward trend over time, but until POD6 was still higher than that in group C. The pain score of the S + M group was lower than that of the S group from 2 h after operation and lasted until POD6. There was no significant difference in pain score between group M and group C. All values are expressed as means ±SD (*n* = 8 per group). * *p* < 0.05 significantly different

In this study, we first constructed the pain model after internal fixation of the tibial fracture. In order to avoid the interference of the behavioral tests on subsequent cellular molecular experiments, the rats that were sacrificed on POD2 were not tested for pain. There were no significant differences in the hemodynamic and respiratory parameters measured during anaesthesia among experimental groups. The duration of operation was controlled within 10-15 min, and the rats woke up within 5 min after the operation. Body weights were not significantly different from baseline (preoperative) to 7 days after surgery in all groups. Before surgery, the Subjective Pain Scale scores of each group was “0”, and there was no difference between the groups. After operation, the rats in the Group S showed obvious protective behaviours such as foot shrinking and avoiding weight bearing. This spontaneous pain-related behaviour occurred 2 h after the operation, and the pain score was significantly higher than that of the Group C (Fig. 2), and lasted until the 6th day (*P* < 0.05). However, the pain score of the group S + M was lower than Group S from 2 h to 6 days after the operation (*P* < 0.05). Group M regimens failed to show significant effects on the pain score. The difference was recovered on the 7th day after operation. These results suggested that the model of acute postoperative pain in rats was established successfully, and morphine analgesia after operation could effectively relieve the pain behaviour of rats.

### Acute postoperative pain was associated with impaired spatial memory after surgery

**Fig. 3:**
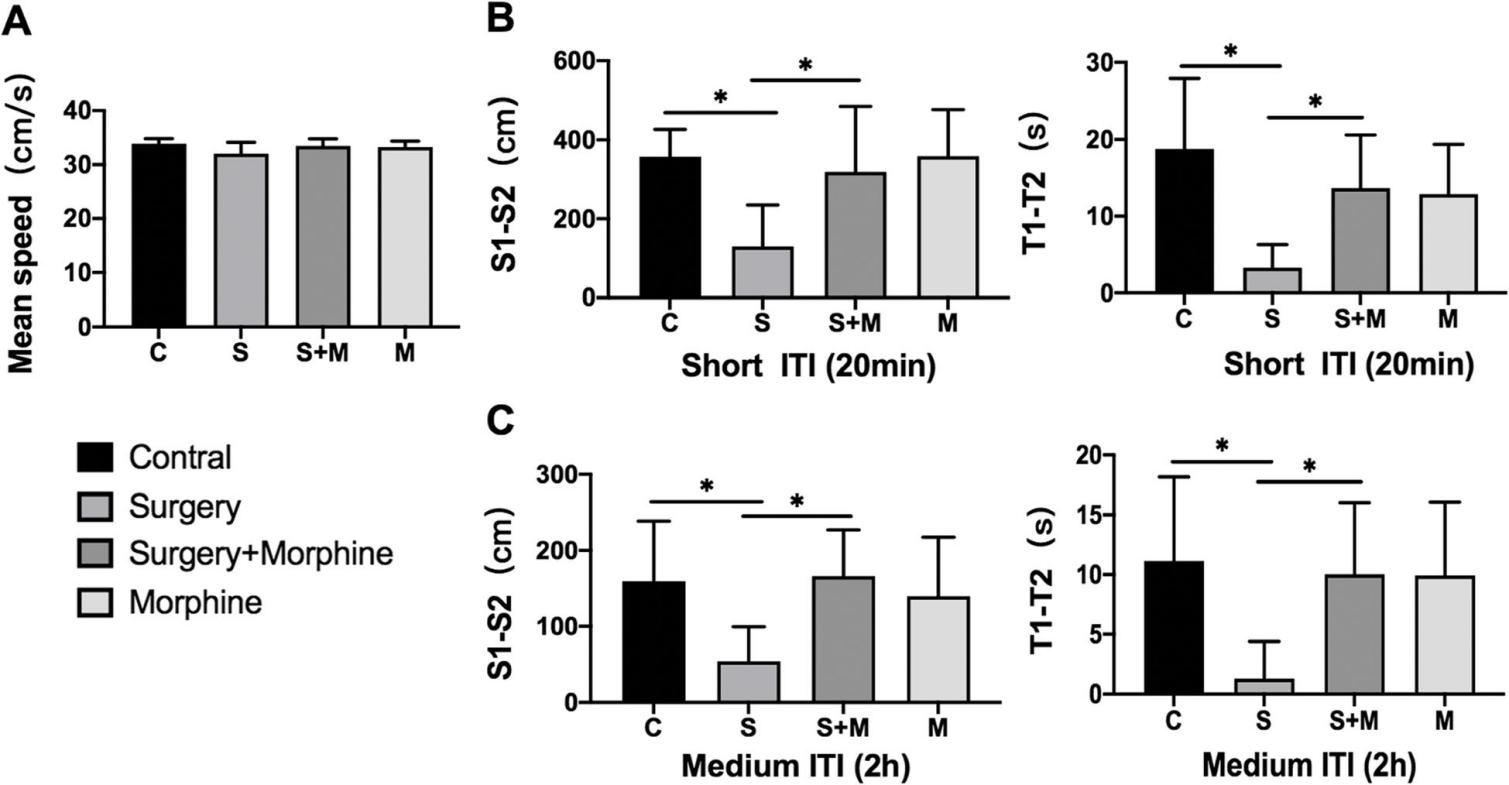
Morris water maze behavioural test results. **a** The swimming speed of the four groups of rats on POD2. **b** The value of path difference and latency difference of ITI 20 min, reflecting short-term memory. **c** The value of the above indicators of ITI 2 h, reflecting the medium long-term memory. All values are expressed as means ±SD, *n* = 8 per group, * *p* < 0.05

The Morris water maze (MWM) is a reliable test to evaluate cognitive functions in rodent animals, but the results can be affected by an animal’s ability to move, especially in terms of speed. Therefore, in this experiment, the swimming speed of rats was first measured on the POD2, so as to exclude the influence of motor ability on behavioural detection (Fig. 3a). The results showed that there was no significant difference in swimming speed between the groups (*P* > 0.05). Then from POD2 to POD7, rats were tested with MWM in two intertrial intervals (ITI 20 min and 2 h), and the difference in path length (S1-S2) and latency (T1-T2) were comprehensively analysed to compare the learning and memory abilities of rats in each group.

The results showed that the values of S1-S2 and T1-T2 in Group S were lower than those in group C at ITI 20 min(*P* < 0.05) (Fig. 3b). The above indexes in Group S + M were significantly higher than those in group S (*P* < 0.05). At ITI 2 h, the difference of the above indexes was similar to ITI 20 min (Fig. 3c). However, there was no significant difference in values of S1-S2 and T1-T2 between M group and C group for ITI 20 min(*P* > 0.05) or ITI 2 h(*P* > 0.05). These results suggested that acute postoperative pain could aggravate the spatial memory impairment in rats, including short-term memory function (ITI 20 min) and medium long-term memory function (ITI 2 h), while postoperative analgesia by morphine could improve this memory injury. Moreover, morphine alone did not affect the memory function of rats, suggesting that morphine improved the spatial memory of rats after operation mainly through its analgesic effect.

### The level of acute postoperative pain was related to the expression of TNF-α in plasma, hippocampus and mPFC

**Fig. 4:**
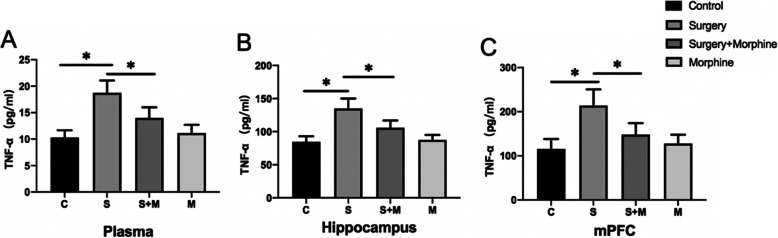
The levels of TNF-α in plasma, hippocampus and mPFC tissues of rats on POD2 (respectively **a**, **b**, **c**). The variation trend of TNF-α content in each group was basically the same in the three tissues, showing that the increase of TNF-α in the S group was inhibited in the S + M group. All values are expressed as means ±SD, *n* = 5 per group, * *p* < 0.05

Peripheral systemic inflammation and CNS inflammation play an important role in the PND, but whether acute postoperative pain exacerbates the above inflammation remains unclear. Therefore, the levels of TNF-α in the hippocampus, mPFC regions and plasma of the rats were determined by ELISA on the POD2. The results showed that on the POD2 (Fig. 4), the levels of TNF-α in plasma, hippocampus and mPFC in group S were significantly higher than in group C (*P* < 0.05), while the level of TNF-α in group S + M was obviously lower than that in group S(*P* < 0.05), and there was no significant difference between group M and Group C (*P* > 0.05). These results suggest that acute postoperative pain is associated with peripheral systemic and CNS inflammatory response, and effective postoperative analgesia can improve these phenomena.

### Microglia cells in hippocampus CA1 regions and mPFC were significantly activated

**Fig. 5:**
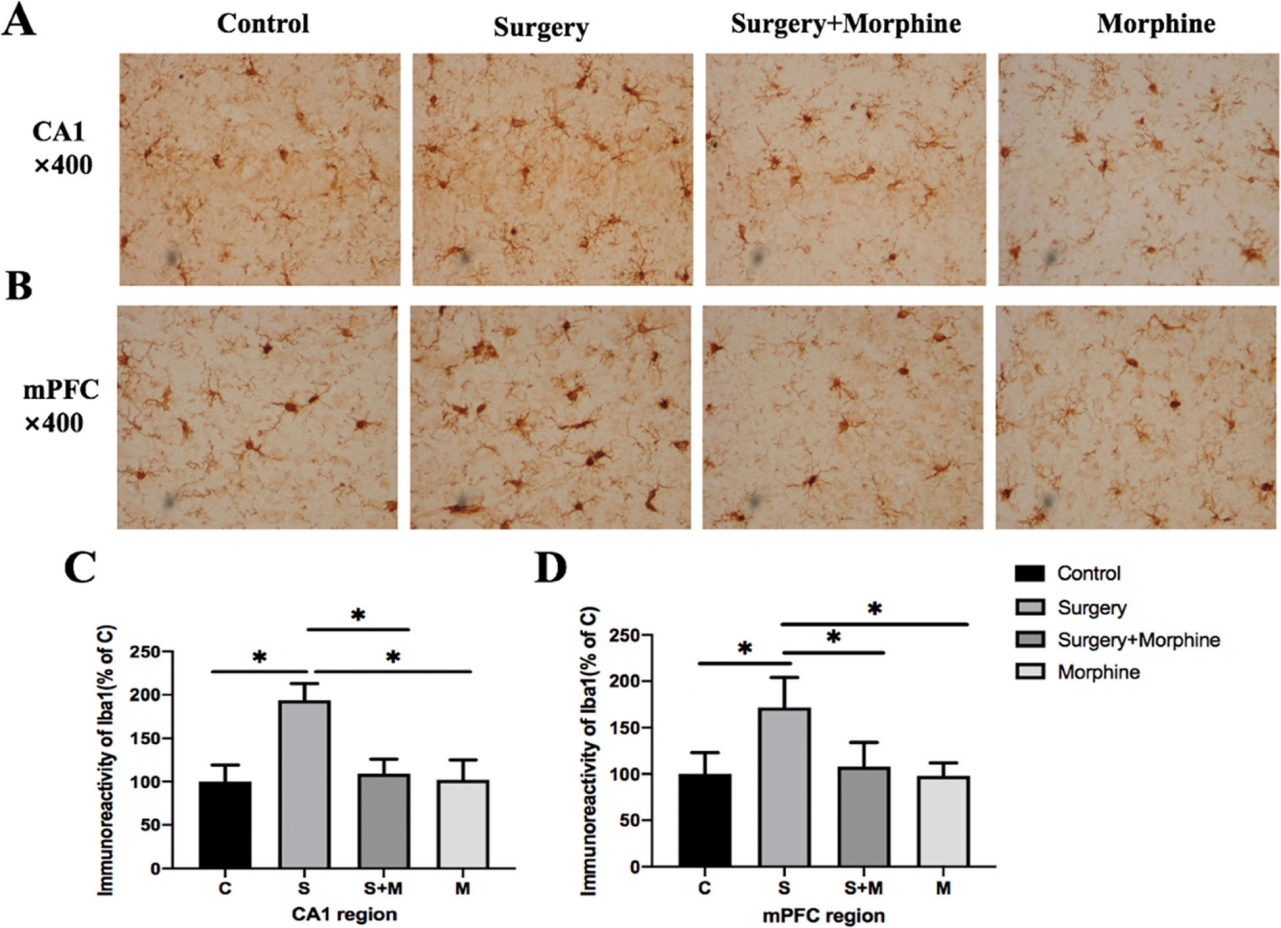
Immunohistochemistry was used to evaluate the activation of microglia cells in CA1 regions and mPFC in each group on POD2. **a**, **b** Representable image of microglia cells expression in CA1 regions and mPFC. Typical morphological changes of activated microglia cells such as short and thick processes, larger cell body volumes and fewer branches. Magnification: × 400 times. **c** and **d** respectively represent the Iba-1(+) cell immunoreactivity quantification in CA1 regions and mPFC between each group. All values are expressed as means ±SD, *n* = 5 per group, * *p* < 0.05

Microglia cells are innate immune cells in the central nervous system and their activation is an important indicator of inflammation in the central nervous system. Therefore, in order to further clarify the relationship between acute postoperative pain and CNS inflammation, we detected the expression of Iba1 (a specific marker of microglia cells) through immunohistochemistry, and quantified the immunoreactivity of Iba1 positive cells in the hippocampal CA1 and mPFC region to evaluate the activation of microglia cells in each group. The activated microglia showed morphological changes such as short and thick processes, larger cell body volumes, and fewer branches (Fig. 5a, b). The quantification results (Fig. 5c) showed that the immunoreactivity of Iba1 (+) cells in the CA1 regions of group S was significantly increased compared with that of group C (*P* < 0.05), and that of group S + M was decreased compared with that of group S(*P* < 0.05). There was no significant difference between group M and group C (*P* > 0.05). In mPFC (Fig. 5d), Iba1 (+) cells in each group showed the same trend as that in the CA1 regions. These results showed that acute postoperative pain was significantly associated with the activation of microglia cells in the hippocampus and mPFC, while sufficient morphine analgesia inhibited the activation of microglia cells.

### C-Fos expression was increased in the hippocampal CA1 region and mPFC

**Fig. 6:**
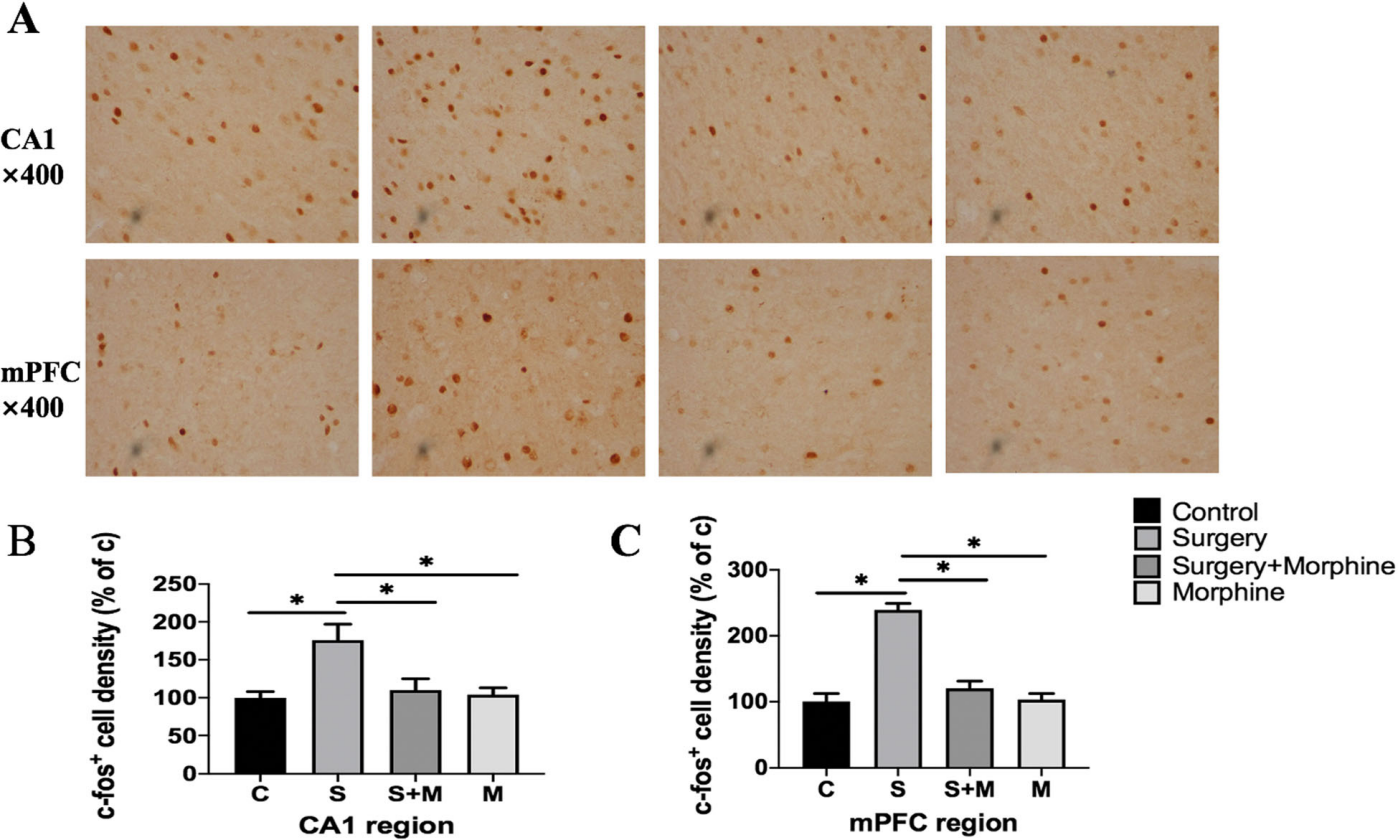
C-fos expression in CA1 regions and mPFC of rats in each group on POD2. Representable image of c-fos(+) expression in CA1 regions and mPFC. Magnification: × 400 times. **b**, **c** Represent the relative quantification of c-FOS protein expression in CA1 regions and mPFC. All values are expressed as means ±SD, *n* = 5 per group, * *p* < 0.05

In order to determine the correlation between the above-mentioned brain dysfunction and acute postoperative pain, we detected the expression of c-FOS protein by immunohistochemistry and compared the neuronal activity of the hippocampus and mPFC between different groups (Fig. 6a). The results showed that the expression of c-Fos in the CA1 regions of Group S was significantly increased compared with that in group C, while that of group S + M was significantly decreased compared with that in group S, and there was no significant difference between group M and Group C (Fig. 6b). In mPFC, the difference was similar to that in the hippocampus CA1 region (Fig. 6c). The above results suggested that increased c-Fos expression in CA1 and mPFC was significantly correlated with acute postoperative pain.

## Discussion

At present, there is no reliable risk stratification method to identify high-risk patients with PND, and many factors are believed to be involved in the occurrence of PND. As a common postoperative complication, acute postoperative pain has been studied largely in terms of its effect on cognitive dysfunction. Although some clinical studies have shown that sufficient postoperative analgesia can reduce the occurrence of PND [4, 22, 23], it can’t be ruled out that analgesics have a direct effect on cognitive function. In this study, we found that spatial memory was significantly impaired in rats with acute postoperative pain. Morphine therapy was effective in reducing pain scores as well as spatial memory impairment. In addition, the non-surgical animals treated with morphine alone failed to show significant differences in spatial memory and brain cytokine levels. These observations suggest that morphine can improve spatial memory in rats after tibial fracture fixation, but does not affect cognitive function in normal rats. Studies have shown that activation of opioid receptors in neurons can induce analgesia, but the opioid receptors have been identified at the mRNA and protein levels in peripheral immune cells (lymphocytes, granulocytes, monocytes, macrophages) and glia in humans, rhesus monkeys, rats or mice [24]. Morphine may affect the function of immune cells, and its role related to immunosuppression in pain modulation has been widely discussed. This study found that morphine can indeed reduce the peripheral and central inflammatory response and the activation of microglia cells, which is consistent with the above viewpoints, but the specific mechanism needs to be further studied.

The pathophysiological mechanism of PND is still unknown, but the CNS inflammatory hypothesis has been widely accepted. Surgical stimulation causes cascaded release of cytokines, which induces systemic inflammation. Studies have shown that plasma levels of pro-inflammatory cytokines such as TNF-α, IL-6 are significantly increased after surgery and associated with the occurrence of PND [6, 25]. As a part of surgical stimulation, acute postoperative pain has been shown to be positively correlated with the intensity of systemic inflammatory response. Ko et al. [9] showed that after hip fracture surgery, patients with more severe pain also had higher levels of TNF-α in their plasma. Animal experiments also found that pain may aggravate cognitive impairment by changing the levels of inflammatory factors such as IL-6 and IL-1β in the plasma of rats [26]. It is generally believed that due to the presence of the blood brain barrier (BBB), the increase of peripheral inflammatory factors does not affect the central nervous system function. However, studies have shown that increased inflammatory cytokines such as TNF-α in plasma can affect the permeability of the BBB through the NF-κB pathway, thereby invading the centre and causing CNS inflammation [27]. The increase of inflammatory factors such as TNF-α in the brain will directly damage neurons, thus affecting brain function; and moreover it will activate microglia in the brain and aggravate the inflammatory response of the central nervous system. Microglia are highly specialised tissue-resident macrophages in the central nervous system (CNS) and the major resident immune cells of the brain [28]. In healthy brains, microglia are ramified and in a resting state, monitoring the local microenvironment and detecting CNS damage [29]. Activated microglia are both neuroprotective and neurotoxic. Studies have shown that the increase of pro-inflammatory cytokines such as TNF-α in the brain can activate microglias, which will further release a variety of pro-inflammatory cytokines after activation. This positive feedback amplifying mechanism of inflammation further aggravates nerve cell damage and thus causes cognitive dysfunction [30–32]. In this study, we observed that acute postoperative pain was indeed associated with plasma TNF-α content, further supporting the idea that acute postoperative pain can aggravate peripheral systemic inflammation. Moreover, TNF-α levels were also increased in the hippocampus and mPFC of the operation group in this experiment, and microglia cells in the above-mentioned brain areas were significantly activated. These results suggest that CNS inflammation occurs in rats with PND, which may be related to acute postoperative pain. Although our results confirm the role of peripheral systemic inflammation and CNS inflammation in PND, and postoperative pain is closely related to inflammation, nonetheless how peripheral systemic inflammation communicates with the central nervous system remains unclear. The specific mechanism needs further study.

Imaging data such as MRI showed that pain activates brain areas including the dorsal-sensory cortex 1 and 2, the medial prefrontal cortex (mPFC), anterior cingulate cortex (ACC), insular cortex (IC), and hippocampus [14, 15]. Among them, the mPFC and hippocampus are responsible for learning and memory function, and their impaired function will significantly impair cognitive function. Preclinical studies also showed that cortical volume decreased in SI (sensory cortex I), SII (sensory cortex II), ACC, mPFC and other brain regions in neuropathic pain rats [33]. Although the overlap of related brain areas provides the anatomical basis for pain exacerbating cognitive impairment, few studies have demonstrated the activation of cognition-related brain regions by postoperative pain at the cellular molecular level. c-Fos is expressed rapidly and instantaneously after neurons are stimulated, and its expression is an indirect marker of neuronal activity, so it can be used as a neural marker of pain [11, 12]. In this study, it was found that the expression of c-Fos increased in the hippocampus and mPFC of rats with acute postoperative pain, and PND occurred in these rats. The above results further confirm that acute postoperative pain can directly affect the function of the hippocampus and mPFC. Moreover, studies have shown that when the brain is subjected to stress or injury, a large amount of c-Fos expression may lead to secondary brain damage and interfere with brain tissue repair [34]. These studies suggest that c-Fos is not only a marker of acute postoperative pain acting on related brain regions, but also can further aggravate the damage to cognitive function. But the specific mechanism needs further study.

There are a few limitations that should be considered. First of all, although we have confirmed that acute postoperative pain can aggravate peripheral systemic inflammation and CNS inflammation, the specific mechanism by which pain affects the intensity of inflammation has not been fully studied. Secondly, the signal communication between peripheral systemic inflammation and CNS inflammation is still a major problem that needs further study. Finally, our model of acute postoperative pain is based on tibial fracture surgery and may not be representative of all complex clinical post-operative pain.

## Conclusions

In conclusion, the results of our study indicated that acute postoperative pain may increase the incidence of perioperative neurocognitive disorders by exacerbating peripheral and central nervous system inflammatory responses; and adequate postoperative analgesia is one of the feasible strategies to prevent PND.

## Data Availability

The datasets used and/or analysed during the current study available from the corresponding author on reasonable request.

## Abbreviations

PND: Perioperative neurocognitive disorders
CNS: Central nervous system
POD2: The second day after surgery
mPFC: The medial prefrontal cortex
ICD: The International Classification of Diseases
Iba1: Ionized calcium binding adaptor molecule 1
MWM: Morris water maze
DMP: Delayed matching-to-place
ITI: The intertrial intervals
ELISA: The enzyme-linked immunosorbent assay
ANOVA: The single-factor analysis of variance
ACC: Anterior cingulate cortex
IC: Insular cortex
